# Better short-term function after unicompartmental compared to total knee arthroplasty

**DOI:** 10.1186/s12891-021-04185-w

**Published:** 2021-04-02

**Authors:** Eric Tille, Franziska Beyer, Kai Auerbach, Marco Tinius, Jörg Lützner

**Affiliations:** 1 University Center for Orthopaedic, Trauma- and Plastic Surgery, University Medicine Carl Gustav Carus Dresden, Technische Universität Dresden, Fetscherstr. 74, 01307 Dresden, Germany; 2 Arthromed Praxisklinik Chemnitz, Chemnitz, Germany; 3 Gelenkzentrum Chemnitz, Chemnitz, Germany

**Keywords:** Unicompartmental knee arthroplasty, Osteoarthritis, Knee, Patient related outcome measures, Total knee arthroplasty, Joint replacement, Short-term outcome

## Abstract

**Background:**

Unicompartmental knee arthroplasty (UKA) is an established treatment option for patients with unicompartmental osteoarthritis (OA). However, strict patient selection is crucial for its success. The proposed advantages include nearly natural knee kinematics, faster rehabilitation and better functional outcomes. Despite the aforementioned facts and it’s proven cost-effectiveness, there are still hesitations for the use of UKA as an alternative to total knee arthroplasty (TKA). Key objectives of this study were therefore to assess clinical and patient-reported outcome (PRO) as well as patient’s satisfaction after medial UKA in comparison to TKA.

**Methods:**

To assess the outcome after UKA we conducted a prospective multi-center study. 116 patients with unicompartmental OA and indication for UKA were included. Overall 54 females and 62 males with an average age of 62.7 years (±9.8) and an average body mass index (BMI) of 29.2 (± 3.7) were recruited. Clinical results and PRO were assessed using the Knee Society Score (KSS). Follow-ups took place 3 months, 1 and 2 years after surgery including clinical examination, radiographs, assessment of PRO and adverse events. Pain and satisfaction was evaluated using a visual analog scale (VAS, 0 (worst) to 10 (best)).

For comparison with TKA a propensity score matched-pair analysis was performed to eliminate confounders. Matching criteria were gender, patient’s age, BMI and comorbidities. A total of 116 matched-pairs were analysed.

**Results:**

There was no revision in the UKA group until 2 years after surgery. Revision rates were higher in the TKA group (0.6%).

Preoperative KSS-Scores were higher within the UKA cohort (*p* <  0.001). After surgical treatment, PROMs displayed a significant improvement (p <  0,001) in both cohorts. Regarding the Knee-Score (Pain, Alignment, ROM) we observed no differences between cohorts after 12 months. The Function-Score demonstrated significantly better results in the UKA cohort (UKA vs. TKA 95 vs 80, *p* <  0.001). Patient satisfaction was also higher in UKA patients (UKA vs TKA 9.0 vs 8.8, *p* = 0.019).

**Conclusion:**

Patients of both cohorts showed high satisfaction after knee arthroplasty. UKA resulted in higher function scores compared to TKA without increased revision rate during short-term follow-up. Therefore, UKA is a good treatment option for unicompartmental OA.

**Trial registration:**

Clinicaltrials.gov, NCT04598568. Registered 22 October 2020 - Retrospectively registered.

## Background

When unicompartmental knee arthroplasty (UKA) was first introduced during the 1970s, there was – as often with new procedures – a high initial failure rate. In order to reduce failures a strict catalogue of indication criteria was established [1]. However the limitations set in this catalogue were so narrow that only few patients qualified for UKA. A study of Stern et al. observed that – used correctly – only 8% of patients met all criteria to be eligible [2]. Currently paradigms are changing, indication criteria are being revised and UKA is used more often [1, 3]. Yet many surgeons still tend to choose total knee arthroplasty (TKA) over UKA due to its proven efficacy, lower revision rates and higher patient satisfaction [4]. However, looking at the number of patients who could benefit of UKA as a less-invasive procedure, this is unfortunate. Especially since there is a high number of patients suffering from isolated unicompartmental osteoarthritis (OA). Satku et al. have presented data leading to the conclusion that up to 20% of patients with OA could sufficiently be treated with UKA [5].

Since there are numerous advantages of UKA such as the less-invasive surgical approach, retention of natural bone stock, preservation of cruciate ligaments, enhanced recovery, a better overall range of motion and more physiological joint kinematics there is a need for readjustment [1, 6, 7]. Especially taking into account that UKA is associated with lower morbidity and mortality rates [8]. In contrast, data from the German Arthroplasty Registry (EPRD) demonstrated that the overall short-term revision rate for UKA was twice as high as for TKA [9]. Furthermore, it has to be mentioned that patients receiving UKA are not always directly comparable to patients receiving TKA. Often patients who receive UKA have less severe OA, better preoperative joint function and less comorbidities. This might contribute to favorable results.

The objective of this study was therefore to assess patient-reported outcome after UKA and comparison with matched patients after TKA.

## Methods

A prospective, multi-center cohort study was performed. Overall, 116 consecutive patients in three centers scheduled for medial UKA were recruited. Criteria for treatment with UKA were advanced isolated medial compartment OA not adequately responding to conservative treatment, a functionally intact anterior cruciate ligament (ACL) and no cartilage degeneration in the lateral and patellofemoral compartment greater than grade 2 according to the Outerbridge classification [10]. 115 patients completed the 2 year follow-up.

The patients were recruited in three separate arthroplasty centers. Three experienced arthroplastic surgeons performed all procedures. All patients received the BalanSys UNI implant system (Mathys AG, Bettlach, Switzerland) with a fixed polyethylene (PE) insert via a limited medial parapatellar approach. A tourniquet was routinely used to reduce bleeding. All components were cemented. The majority of 110 patients were treated in-hospital. In six cases the surgery was performed as an out-patient procedure. All patients underwent a standardized rehabilitation program with full weight-bearing. Initially crutches were used for mobilization as needed. The patient cohorts displayed an equal distribution with regards to gender (54 females, 62 males). The average patient age was 62.4 years (57.7; 70.8). Mean body mass index (BMI) was 29.2 kg/m^2^ (26.9; 31.9). Mean cut-sew time was 72 min (65.0; 78.0).

Patients were assessed preoperatively, as well as 3, 12 and 24 months after surgery using validated patient reported outcome measures (PROMs). The evaluated PROMs included the Knee injury and osteoarthritis outcome score (KOOS) consisting of 5 subscales (pain, symptoms, satisfaction, activities of daily living and quality of life) [11, 12]. According to Roos et al. an alteration of 8 points or more represents a clinically significant change [12]. Furthermore the Knee Society Score (KSS) [13] and the subjective pain levels measured by visual analogue scale (VAS, 0 (no pain) to 10 (worst pain)) were evaluated. VAS was assessed during rest and under load. In addition, we recorded patient’s satisfaction also using a VAS from 0 (very dissatisfied) to 10 (very satisfied). The KSS which includes the Knee Score (pain, alignment, ROM, stability) and the Function Score (walking distance, stairs, use of walking aids) [13] was used to assess the functional outcome. According to Lizaur-Utrilla et al. a change of 9 points in the Knee score and 10 points in the Function score can be regarded as clinically important change [14]. Results can be graded in the following categories: 100 – 80points = excellent, 70–79 = good, 60–69 = fair, < 60 = poor results. At all scheduled follow-ups radiographic evaluation regarding positioning of the implant and signs of loosening took place. Furthermore, the mechanical and anatomical axis as well as implant alignment (medial proximal tibial angle (MPTA) and tibial posterior slope) were measured. All radiographs were assessed by one investigator.

For comparison with TKA a propensity score matched-pair analysis was performed to eliminate confounders. Patients from the UKA cohort were matched to patients with a TKA from the local TKA registry. In this registry, the KSS and adverse events were assessed prospectively at three timepoints: before surgery, 3 and 12 months after surgery. At the 12 months follow-up satisfaction regarding the result of the surgery was assessed. Matching criteria were gender, patient’s age, BMI and comorbidities (ASA-score). A total of 116 matched pairs were analysed. Matching was carried out using R software, package “matching”. A propensity score matching for the nearest neighbour with replacement was performed with exact matching for the variables gender and ASA score and propensity matching for age and BMI.

### Statistical analysis

All data was collected in a database. SPSS release 24 for Windows (SPSS Inc., Chicago, IL, USA) was used for statistical analysis. Data was analysed for normal distribution with Kolmogorov-Smirnov test. Data is presented as median (25th percentile; 75th percentile) for continuous variables and absolute (relative) frequencies for categorical variables. Comparisons between groups were based on Mann-Whitney-U test for continuous variables and on chi-square tests for categorical variables, respectively. Results of all significance tests were summarized as *p* values. The minimum level of significance accepted was *p* <  0.05.

## Results

There was no revision in the UKA group until 2 years after surgery. Revision rates were higher within the whole TKA group (0.6%).

After surgical treatment all evaluated PROMs displayed a significant improvement in both cohorts compared to the preoperative status. Evaluation of the KSS after 12 months displayed an additional significant improvement for the function score within the UKA cohort (UKA 95 vs 80 TKA, *p* <  0.001). The knee score showed no statistic difference between the cohorts (UKA 90 vs 94 TKA, *p* = 0.184). The better function score within the UKA cohort was caused by an improved walking distance (66.1%with unlimited walking distance in UKA vs 31.9% in TKA) and the better ability to climb stairs (64.3%without impairment in UKA vs 31% in TKA) (Table 1).

**Table 1:** Patient-reported Outcome for TKA and UKA

	TKA	UKA	***p***-value
**Knee-Score**			
preoperative	37 (29; 49)	51 (44; 60)	< 0.001
12 months	94 (79; 96)	90 (84; 94)	0.184
difference	49 (35; 61)	36 (27; 45)	< 0.001
**Function Score**			
preoperative	50 (50; 60)	60 (50; 70)	0.003
12 months	80 (60; 90)	95 (80; 100)	< 0.001
difference	20 (10; 30)	30 (20; 40)	< 0.001
**Satisfaction**			
12 months	8.8 (8.0; 9.5)	9 (8.0; 10.0)	0.019

Comparison of Patient-reported Outcome for TKA and UKA. Values are given as median (25th percentile; 75th percentile)

**Fig. 1:**
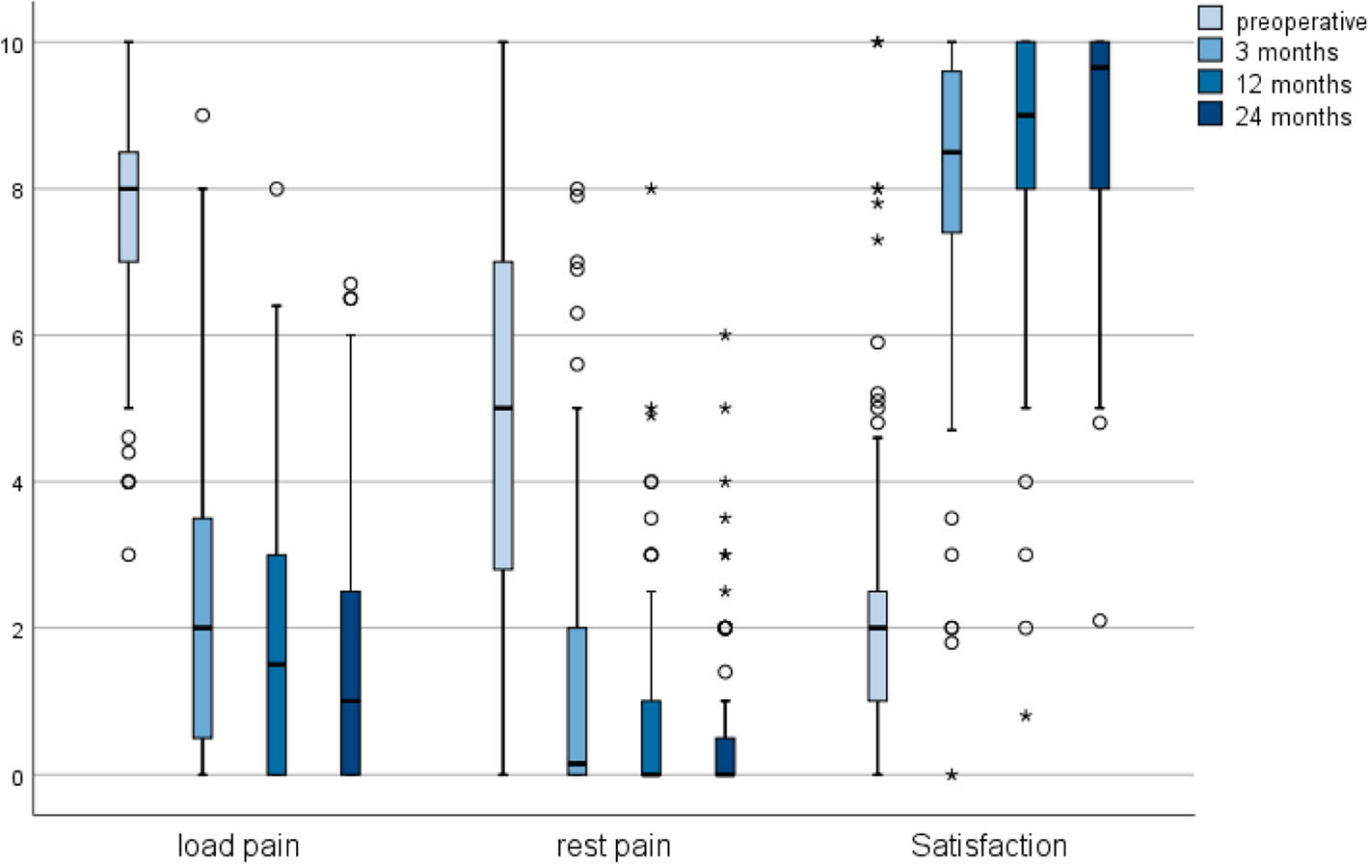
Subjective pain levels and satisfaction according to visual analogue scale at each given Follow-Up for UKA cohort. UKA = Unicompartimental Knee Arthroplasty

Within the UKA cohort we observed a significant pain reduction in resting patients at the 3 month follow-up (FU). There was a further significant improvement between the 3 month and 12 month FU. This was accompanied by a rise in patient’s satisfaction levels. Recorded pain and satisfaction levels are displayed in Fig. 1.

**Fig. 2:**
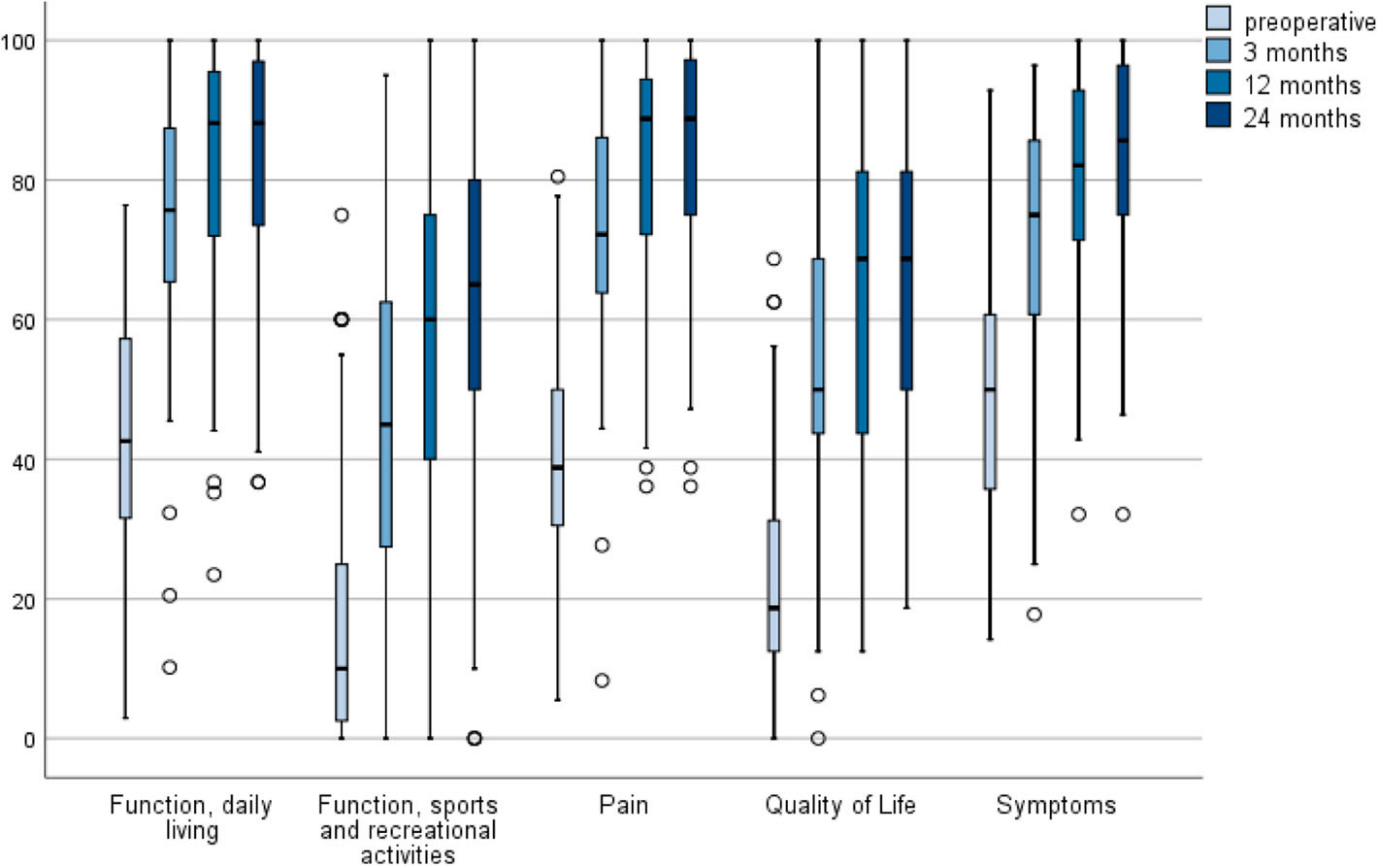
KOOS subscales within the UKA cohort during follow up. KOOS = Knee Osteoarthritis Outcome Score

KOOS was evaluated only within the UKA cohort. There was an improvement in all sub-categories (pain, symptoms, function daily living, function sports and recreational activities, quality of life). The most significant improvement was observed in the subcategories “Quality of Life” (pre 18.7 (12.5; 31.2) vs 68.7 (43.7; 81.2) at 12 months) and “Participation in sports and recreational activities” (pre 10 (1.3; 25) vs 60 (40; 76.3) at 12 months) (Fig. 2). While major improvements were observed comparing the preoperative status and the outcome measurements at 3 and 12 months after surgery, there was only little further improvement between the 12 and 24 month FU.

For most of the evaluated PROMs we found no differences between the three study centers. Only for single items (KOOS: ADL, Symptoms and KSS: Function Score) we observed slight differences at the 24 month FU.

Radiographic and demographic findings as well as results of the PROMs are summarized in Table 2.

**Table 2:** Comparison between study centers

	Center A	Center B	Center C	p-value
*Demographic findings*				
*gender*				
*Female*	12	32	11	0.210
*Male*	22	27	13	
*age*	59.5 (53.0; 64.2)	65.0 (60.5.; 73.6)	61.5 (56.2; 64.7)	0.001*
*BMI*	28.9 (27.4; 32.8)	29.4 (26.9; 31.9)	29.1 (26.7; 33.0)	0.976
*Cut-sew time*	78.5 (72.0; 84.0)	72.0 (68.0; 76.0)	60.0 (60.0; 60.0)	< 0.01*
*Radiographic findings*				
*Mechanical alignment (− Varus, + Valgus)*	−4.0 (−5.0; − 2.6)	−2.7 (− 3.9; − 1.3)	− 4.8 (− 7.4, − 3.4)	0.019
*Anatomical axis (neutral = 186°)*	182.1 (181; 183.4)	183.3 (182.1; 184.7)	181.2 (178.6; 182.6)	0.019
*Medial proximal tibia angle (MPTA)*	86.6 (84.6; 88.6)	87.5 (86.3; 89.0)	86.3 (85.6; 88.0)	0.105
*Posterior Tibial Slope*	86.8 (85.2; 88.6)	83.8 (81.7: 85.0)	87.2 (84.9; 81.7)	< 0.01
*Patient reported outcome*				
*Knee Society Score*				
*Knee Score*				
*1 years*	88.5 (75.0; 93.0)	90.0 (86.0; 94.0)	88.0 (75.0; 95.0)	0.414
*2 years*	90.0 (80.0; 94.0)	92.0 (86.0; 95.0)	86.5 (73.0; 94.0)	0.048*
*Function Score*				
*1 year*	100 (90.0; 100)	97.5 (80.0; 100)	90.0 (80.0; 100)	0.513
*2 years*	100 (90.0; 100)	90.0 (80.0; 100)	100.0 (90.0; 100)	0.054
*Total Knee Score*				
*1 year*	177.5 (165.0; 192.0)	185.5 (161.0; 191.0)	184.0 (168.0; 186.0)	0.584
*2 years*	182.5 (179.0; 193.0)	183.0 (166.0; 191.0)	178.0 (163.0; 194.0)	0.512
*KOOS*				
*Pain*				
*1 year*	91.6 (75.0; 97.2)	88.8 (69.4; 94.4)	83.3 (77.7; 91.6)	0.513
*2 years*	88.8 (72.2; 97.2)	91.6 (77.7; 97.2)	77.7 (69.4; 86.1)	0.054
*Symptoms*				
*1 year*	82.1 (67.8; 92.8)	85.7 (75.0; 92.8)	78.5 (71.4; 85.7)	0.197
*2 years*	82.1 (75.0; 92.8)	89.2 (78.5; 96.4)	78.5 (64.2; 89.2)	0.008*
*Function, daily living (ADL)*				
*1 year*	92.6 (72.0; 98.5)	89.7 (72.0; 95.5)	86.7 (70.5; 92.6)	0.247
*2 years*	89.7 (75.0; 98.5)	91.1 (73.5; 97.0)	80.8 (72.0; 85.2)	0.031*
*Function, sports and recreation*				
*1 year*	50.0 (30.0; 70.0)	65.0 (35.0; 80.0)	65.0 (50.0; 80.0)	0.243
*2 years*	65.0 (40.0; 75.0)	65.0 (50.0; 85.0)	67.5 (55.0; 75.0)	0.918
*Quality of Life (QoL)*				
*1 year*	68.7 (43.7; 87.5)	71.9 (43.7; 81.2)	68.7 (50.0; 75.0)	0.875
*2 years*	68.7 (50.0; 81.2)	75.0 (50.0; 93.7)	68.7 (50.0; 75.0)	0.368
*Load pain (VAS)*				
*1 year*	0.7 (0.0; 3.4)	1.5 (0.0; 3.0)	2.0 (1.0; 3.0)	0.163
*2 years*	0.4 (0.0; 2.4)	1.5 (0.0; 2.0)	2.5 (1.0; 4.0)	0.007
*Satisfaction*				
*1 year*	10 (8.6; 10)	9.0 (8.0; 10)	9.0 (7.0: 10)	0.108
*2 years*	10 (8.3; 10)	9.8 (8.5; 10)	9.0 (8.0;10)	0.076

Summary of patient-related outcome measures, functional findings and demographic parameters. * = statistically significant result, *p* < 0.05

## Discussion

Both, UKA and TKA increase mobility, improve function, reduce pain levels and therefore raise patient’s satisfaction. Yet there are slight differences in outcome.

While the KSS Knee score displayed no difference between UKA and TKA cohort, Function score was better within the UKA cohort. The function score sums up the criteria of activities of daily living. Patients within the UKA cohort showed in comparison to matched TKA patients a clear advantage. Especially the ability to climb stairs and the walking distance were improved in the UKA group. This is consistent with previous studies [15]. Pain seems to be reduced effectively by both, UKA and TKA.

As shown in Table 2 there were minor differences regarding the patient demographics in the three study centers (age, cut-sew-time, etc.). Furthermore, the evaluation of the radiographic images displayed slight variations of the leg axis and implant alignment. Despite these differences, the PROMs at the 12 and 24 month follow-up did not show a significant difference between the study centers. This demonstrates that the use of this specific UKA implant in medial OA results in consistent outcomes independent of differences caused by hospital, surgeon and patient-related factors.

These findings are supported by numerous recent studies. Table 3 gives an overview of recent publications comparing UKA and TKA in OA highlighting various aspects. While study designs, size of patient population and implants vary, the results tend to favor UKA over TKA where applicable. A drawback however of many studies is the fact, that usually UKA patients have less severe OA, better function prior to surgery and less comorbidities. In these studies, the favorable result might not solely be caused by the operative treatment. It is therefore most important to compare matched patients to reduce possible confounders. Dalury et al. presented a study evaluating the outcome of UKA in comparison to TKA within the same patient. While there was no difference in PROMs, the postoperative ROM was better within the UKA cohort. When asked which implant the patients preferred 12 out of 23 voted for the UKA. No patient favored TKA [32].

**Table 3:** Recent studies comparing UKA and TKA

Author	Year	study	Study design	Result
Tu et al. [16]	2020	121 UKA vs 35 TKA in theIsolated lateral OA, mean FU5.3 years	retrospective,monocenter	better postoperative Oxford Knee Score, Hospital for Special Knee Surgery score, range of motion, shorter length of hospitalization, and higher satisfaction rate in UKA
Liebensteineret al. [17]	2020	112 UKA vs. 330 TKA in medialOA	retrospective,multicenter (registrydata)	no significant differences between WOMAC and early or late ROM
Blevins et al.[18]	2020	150 UKA vs 150 TKA	retrospective,monocenter, matched-pair	UKA patients had less postoperative pain, earlier return to work, andhigher KSS
Hauer et al.[19]	2020	35 UKA vs 35 TKA, mean FU2.3 years	retrospective,monocenter, matched-pair	UKA better regarding Tegner Activity Scale and ROM, better results insubscales of Short-Form 36
Harbourneet al. [20]	2019	420 UKA vs 575 TKA, FU 12 months	prospective,longitudinal cohortstudy, multicenter	UKA patients more likely to return to desired activity
Lum et al.[21]	2018	650 UKA vs 1300 TKA inseverely obese patients, meanFU 2.3 years	retrospective	UKA with equal survivorship with substantially fewer reoperations,reduced deep infection, and less perioperative complications, severelyobese patients had improved Knee function scores
Miglioriniet al. [22]	2018	3254 UKA vs 10.649 TKA	Meta-analysis	UKA with better clinical and functional outcome, yet reducedsurvivorship
Goh et al.[23]	2018	160 UKA vs 160 TKA, patientsyounger than 55, mean FU 7 years	prospective,multicenter, matched-pair	no significant difference in Knee Society Score, Oxford Knee Score,and Short-Form 36; greater ROM in short-term (2 years)
Lombardiet al. [24]	2018	UKA vs TKA revision, *n* = 193,mean postoperative interval4.8 years	retrospective,monocenter	Re-revision rates after UKA revision equal to primary TKA and lowercompared to Re-revision TKA
Siman et al.[25]	2017	120 UKA vs 188 TKA, patientsolder than 75 in medial OA	retrospective,monocenter	UKA with shorter operative time and hospital stay, lowerintraoperative blood loss / transfusions, greater postoperative range ofmotion, higher level of activity at time of discharge, no difference inpostoperative KSS, or 5-year survivorship
Kulshresthaet al. [8]	2017	40 UKA vs 40 TKA, comparisonon early medial OA, FU 2 years	prospective,randomized,monocenter	UKA with similar improvement in patient-reported outcomes, function,and performance; UKA with shorter hospital stay and fewercomplications
van der Listet al. [26]	2017	166 UKA vs 63 TKA,comparison in medial OA,mean FU 3 years	retrospective,monocenter	better functional outcome in UKA, especially in younger patients andfemales
Lum et al.[27]	2016	201 UKA vs. 189 TKA, mean FU5.5 years	retrospective,monocenter	UKA with higher postoperative Knee Function Score, no differences inROM, revision rates without statistical differences
Shankar et al.[28]	2016	64 UKA vs 64 TKA, cost analysis	retrospective,monocenter, matched-pair	UKA with shorter operative time, hospital stay, lower transfusion rates,earlier discharge, overall lower direct and total cost
van der Listet al. [29]	2016	48 UKA vs 34 TKA, comparisonin lateral OA, mean FU 2.8 years	retrospective,monocenter	UKA with superior short-term functional outcome (WOMAC), especiallyin young patients and females
Fabre-Aubrespyet al. [30]	2016	101 UKA vs 101 TKA, patientsolder than 75, FU 5 years	retrospective,monocenter, matched-pair	UKA with better KSS, KOOS and Forgotten Knee score, similar 16 yrsurvivorship
Schwab et al.[31]	2015	105 UKA vs 105 TKA	retrospective,monocenter, matched-pair	lower blood loss and transfusion rates in UKA

*UKA* Unicompartmental Knee Arthroplasty, *TKA* Total Knee Arthroplasty, *OA* Osteoarthritis, *FU* Follow-Up, *WOMAC* Western Ontario and McMaster Universities Osteoarthritis Index, *ROM* Range of Motion, *KSS* Knee Society Score

The results of our study with better function in the UKA group are consistent with prior matched-pair studies. Blevins et al. observed that UKA patients suffered less postoperative pain, achieved a higher KSS and were able to return to their workplace sooner than patients receiving TKA [18, 27]. Hauer et al. found an improvement in the Tegner Activity Scale (TAS) and a better ROM [19]. Even though there is a growing number of studies underlining the better clinical and functional outcome of UKA, there are also reports showing no benefit of UKA over TKA. However, these studies demonstrate at least equal functional results [17, 23]. Further reported advantages of UKA include a shorter length of hospital stay, a lower blood loss and accordingly lower transfusion rates as well as lower overall costs [16, 25, 28, 31].

While there seem to be many reasons emphasizing the beneficial use of UKA, there has been one main reason against its use: the potentially higher revision rates. Data from the German Arthroplasty Registry (EPRD) demonstrated an increased early failure rate after 12 months. After 4 years the failure rate had doubled compared to TKA [9]. Higher failure rates however seem to be related to low-volume hospitals [33–35]. Recent studies observed – a strict patient selection provided - an equal short- and mid-term survivorship for UKA and TKA [21, 24, 25]. The studies of Fabre-Auberspy et al. even found an equal 16-year survivorship of UKA and TKA [30]. Lum et al. demonstrated an equal survivorship with substantially fewer revisions, reduced deep infections and less perioperative complications in UKA compared to TKA in an average FU of 2.3 years [21]. Even in patients, which had not been considered as “ideal patients” for UKA good results have been reported.

Despite these optimistic findings there are still reports pointing in another direction. In a recent meta-analysis Migliorini et al. described a better clinical and functional performance of UKA while observing a reduced survivorship [22]. Berend et al. reported a reduced 2-year UKA survival rate of 78.8% in patients with obesity [36]. Data of the Finnish Arthroplasty Registry attribute a worse survivorship of UKA compared to TKA after 5- and 10-year FU [37]. In order to tackle these issues Murray et al. emphasize the importance of appropriate indication criteria and the necessary experience of the executing surgeon [4]. Previous publications suggest that a higher number of performed UKA procedures is directly correlated to reduced revision rates. Data of the National Joint Registry of the United Kingdom (NJR) show a decreasing revision rate if UKA is utilized in up to 20% of arthroplastic cases [38].

Most important, however, is the patient selection, since not all patients and age groups benefit equally from treatment with UKA [25, 30]. In the past Kozinn and Scott outlined contraindications for the use of UKA in OA including but not limited to obesity, a high level of activity and high preoperative pain levels [39]. Many of these criteria have been revised since. Yet there is still no consensus regarding indication criteria for UKA. Recent studies show that especially younger patients and females tend to benefit over proportionally from UKA displaying a higher ROM, faster return to work and higher PROM scores [26, 29]. However UKA does not seem to be limited to these patients. As Seng et al. showed, even patients with severe deformities reaching beyond the standard indications for UKA can achieve good functional results if the correct mechanical alignment is restored [40]. The aforementioned study by Lum et al. demonstrated an additional benefit regarding functional outcome in severely obese patients [21]. And even elderly patients over 75 years have displayed a superior functional outcome [30]. However, these results are being controversially discussed. In an effort to establish an indication tool, Antoniadis et al. developed a scoring system (Unicompartmental Indication Score, UIS), trying to predict the expected postoperative result depending on numerous independent variables (i.e. age, cause of symptoms and Kellgren-Lawrence Grade). While there was no correlation regarding single demographic factors, the postoperative PROMs and satisfaction were significantly higher in patients with a high preoperative UIS. Patients with a low UIS on the other hand reported less beneficial results [41]. This leads the authors to the conclusion that not a single factor but rather a combination of several parameters affects the outcome of UKA.

For our study we acknowledge some limitations. Since – in contrast to the UKA cohort – the TKA cohort was not recruited multicentric there might be a bias due to the lower variety in patient population and number of surgeons. Furthermore, not all PROMs were evaluated at all given FU time points for the TKA cohort. Therefore, the results of the UKA cohort display a more detailed picture of the recovery process after surgery.

## Conclusions

This study demonstrated overall good short-term results of fixed-bearing medial UKA. This resulted in better function after medial UKA compared to TKA in matched patients with primary knee OA. UKA should therefore be considered more often in treatment of unicompartmental knee OA if the surgeon is experienced in this technique.

## Data Availability

The datasets used and/or analysed during the current study are available from the corresponding author on reasonable request.

## Abbreviations

ACL: Anterior cruciate ligament
ADL: Activities of Daily living
BMI: Body-Mass Index
FU: Follow up
KFS: Knee Function Score
KOOS: Knee injury and osteoarthritis outcome score
KSS: Knee society score
MPTA: Medial proximal tibial angle
NJR: National Joint Registry
OA: Osteoarthritis
PROM: Patient related outcome measurement
QOL: Quality if living
ROM: Range of motion
TAS: Tegner Activity Scale
TKA: Total knee arthroplasty
UIS: Unicompartmental Indication Score
UKA: Unicompartmental knee arthroplasty
VAS: Visual analogue scale
WOMAC: Western Ontario and McMasters Universities Osteoarthritis Index
